# A Novel Dual-Band Circularly Polarized Wearable Antenna

**DOI:** 10.3390/mi15050588

**Published:** 2024-04-28

**Authors:** Yu Dong, Hong Lu, Xing Chen

**Affiliations:** College of Electronic and Information Engineering, Sichuan University, Chengdu 610064, China; yudong0327@163.com (Y.D.); luh@crirp.ac.cn (H.L.)

**Keywords:** circular polarization (CP), dual-band, wearable antenna, monopole antenna, SAR

## Abstract

A circularly polarized wearable antenna operating in the 2.45/5.8 GHz ISM dual bands is proposed, which consists of a coplanar waveguide-fed rectangular monopole antenna and two parasitic branches. The monopole rectangular radiation patch can generate 2.45 and 7 GHz frequency bands and has linear polarization characteristics. By adding L-shaped parasitic branches and L-like grounded branches on both sides of the monopole, the 7 GHz operating frequency band can be moved to the 5.8 GHz frequency band, and circular polarization characteristics can be achieved in both the 2.45 and 5.8 GHz frequency bands. The size of the antenna is 48.7 mm × 42.1 mm × 1.016 mm. The simulated −10 dB impedance bandwidths of the antenna are 1.8–2.66 GHz and 5.48–6.98 GHz, respectively. The 3 dB axial ratio bandwidths are 2.34–2.67 GHz and 5.58–6.39 GHz, respectively, and it has dual-band circular polarization characteristics. In addition, the radiation characteristics of the antenna and its safety performance near the human body were analyzed. The antenna prototype has been constructed, and the measurement and simulation results have good consistency. The proposed antenna is suitable for application in wearable devices.

## 1. Introduction

In recent years, wearable devices for wireless body area networks (WBANs) have emerged continuously, and have been widely used in fields such as leisure and entertainment, medicine and healthcare, and the military [1]. In wearable devices, wearable antennas play a mediating role in data transmission, and the performance of the antenna directly affects the performance of the entire system. Due to the complex electromagnetic characteristics of the human body, wearable antennas are required to have certain performance requirements, such as having a low profile and small size, and being broadband, multi-band, capable of circular polarization, flexible, and safe [2,3].

To make the antenna have a low profile, wearable antennas usually take the form of microstrip patch antennas [4,5,6] and monopole antennas [7,8,9]. The wearable antennas mentioned above only focus on wearability, and most of them are linearly polarized antennas. Linearly polarized antennas have difficulties in polarization matching, severe multipath reflection, and poor anti-interference abilities, which can no longer meet the application needs of various communication scenarios. However, circularly polarized antennas are gradually being favored by researchers due to their low polarization loss and good ability to solve polarization mismatch and multipath reflection problems between antennas [10,11,12,13,14]. In Ref. [10], a graphene-based low-profile wearable antenna was designed, and the authors used an anisotropic artificial magnetic conductor array combined with unipolar radiation patches to achieve the right-hand circular polarization of the wearable antenna, with 3 dB axial ratio bandwidths ranging from 5.75 to 5.83 GHz. This antenna also has flexibility and a low specific absorption rate. Reference [11] designed the first circularly polarized button antenna with omnidirectional radiation, which consists of a button, a supporting shaft, a flexible dielectric plate, a fabric layer, and a ground plane. The actual diameter is only about 1 cm, which is consistent with the actual button size. This antenna achieves circular polarization by generating a 90° phase difference through an electric dipole and three radiation arms, with an impedance bandwidth range of 5.68–5.91 GHz and a circular polarization axis ratio bandwidth range of 5.72–5.91 GHz. Reference [12] designed a miniaturized ultra-wideband circularly polarized antenna using denim fabric as the substrate. In the design, the single-feed method is used to achieve preliminary circular polarization by improving the octagonal strip monopole antenna and asymmetric L-shaped ground. In addition, crossed short rods and truncated grooves are used to widen the axial ratio, and ultra-wideband circular polarization is realized. The impedance bandwidth range of this antenna is 3.09–11 GHz, and the axial ratio range is 3.4–10 GHz. Reference [13] proposes a two-element circularly polarized MIMO antenna. The designed MIMO antenna consists of two sickle-shaped patches and a common ground plane. Each antenna element is excited by a microstrip line feed, and an inverted L-shaped strip is supported on the ground to achieve circular polarization. The impedance and axial bandwidths of the antenna can cover 3.6–13 GHz and 5.2–7.1 GHz, respectively. Reference [14] proposes a wearable antenna with dual bands and dual polarization. It consists of a square radiation patch with chamfered edges and an artificial magnetic conductor (AMC) plane. The antenna has good linear polarization in the 2.45 GHz frequency band and circular polarization in the 1.575 GHz frequency band. Most of the above-mentioned circularly polarized antennas are single-band antennas, and their functions are limited.

With the rapid development of smart wearable devices, wearable antennas operating in dual or multiple bands have begun to attract attention due to their practicality and multi-functionality. The application of dual-band circularly polarized antennas to wireless body area network systems can greatly reduce the complexity and cost of these systems and reduce the size of the systems [15]. A dual-circularly polarized textile antenna operating in the 3.5 GHz and 5.8 GHz bands was designed in Ref. [15]. This antenna consists of a dual-band monopole antenna and a polarized rotating artificial magnetic conductor (PRACM). With the PRACM, double CP radiation is achieved, where the left-hand CP is generated in the low-frequency band and the right-hand CP is achieved in the high-frequency band. The −10 dB impedance bandwidths of the antenna in the dual frequency bands are 11.7% and 9.1%, respectively, and the 3 dB axial ratio bandwidths are 2.0% and 8.2%, respectively. The antenna mainly relies on polarized rotating artificial magnetic conductors to achieve circular polarization, and its structure is relatively complex. At present, the research on dual-band circularly polarized wearable antennas is still in its infancy, so a relevant design is necessary for the development of wireless body area network systems.

In this paper, we propose a miniaturized circularly polarized antenna fed by a coplanar waveguide (CPW) operating in the 2.45/5.8 GHz ISM dual bands (2.400–2.4835 GHz and 5.725–5.875 GHz), utilizing monopole radiation patches and left and right parasitic branches to achieve dual-band circularly polarized characteristics.

## 2. Antenna Design

### 2.1. Antenna Configuration

**Figure 1:**
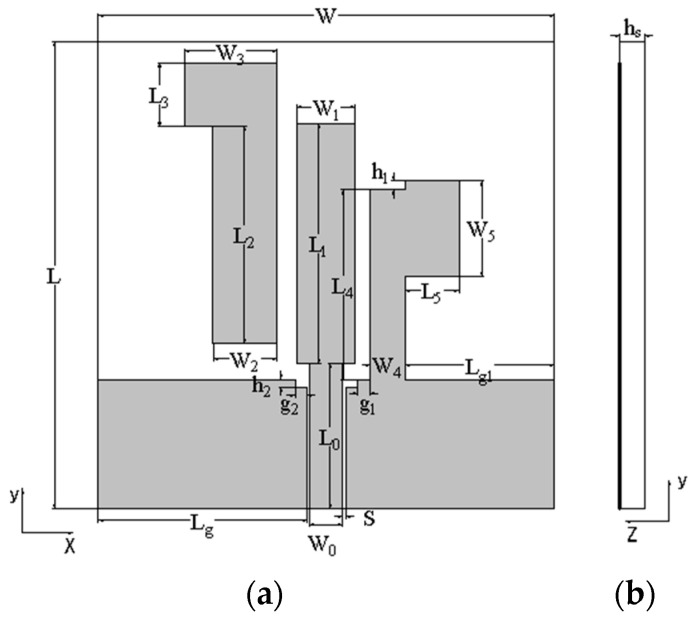
The geometry of the proposed monopole antenna: (**a**) front view; (**b**) side view.

The configuration of the proposed dual-band dual-circularly polarized antenna is illustrated in Figure 1. The antenna substrate uses a double-sided Taconic TRF-45 board with a thickness of 1.016 mm, which has a relative dielectric constant of 4.5 and a loss tangent of 0.0035. The antenna consists of a coplanar waveguide-fed monopole antenna and two parasitic branches. The width of the coplanar waveguide feeder is W0, the length is L0, the gap between the coplanar waveguide feeder and the ground plane is s, and the upper end is connected to a monopole radiator. To the left of the monopole, an L-shaped parasitic branch structure is indirectly coupled to it. To the right of the monopole, an L-like ground branch is used to improve the axial ratio bandwidth of the circularly polarized antenna. To further adjust the impedance-matching performance of the antenna, two rectangular cutouts were made above the CPW ground on both sides of the feeder. The proposed dual-band dual-circularly polarized antenna was simulated and optimized using high-frequency electromagnetic simulation software, and its final dimensions are shown in Table 1.

### 2.2. Antenna Evolution

**Figure 2:**
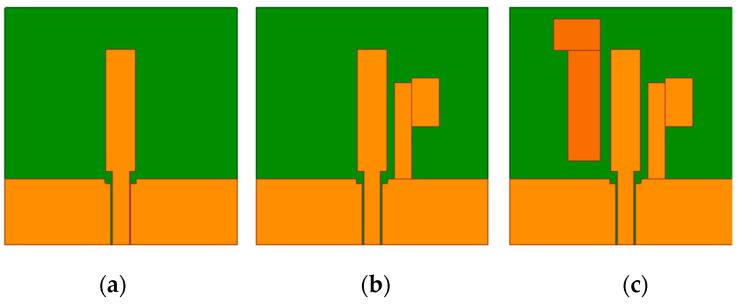
Evolution of the proposed design. (**a**) Ant1; (**b**) Ant2; (**c**) Ant3.

**Figure 3:**
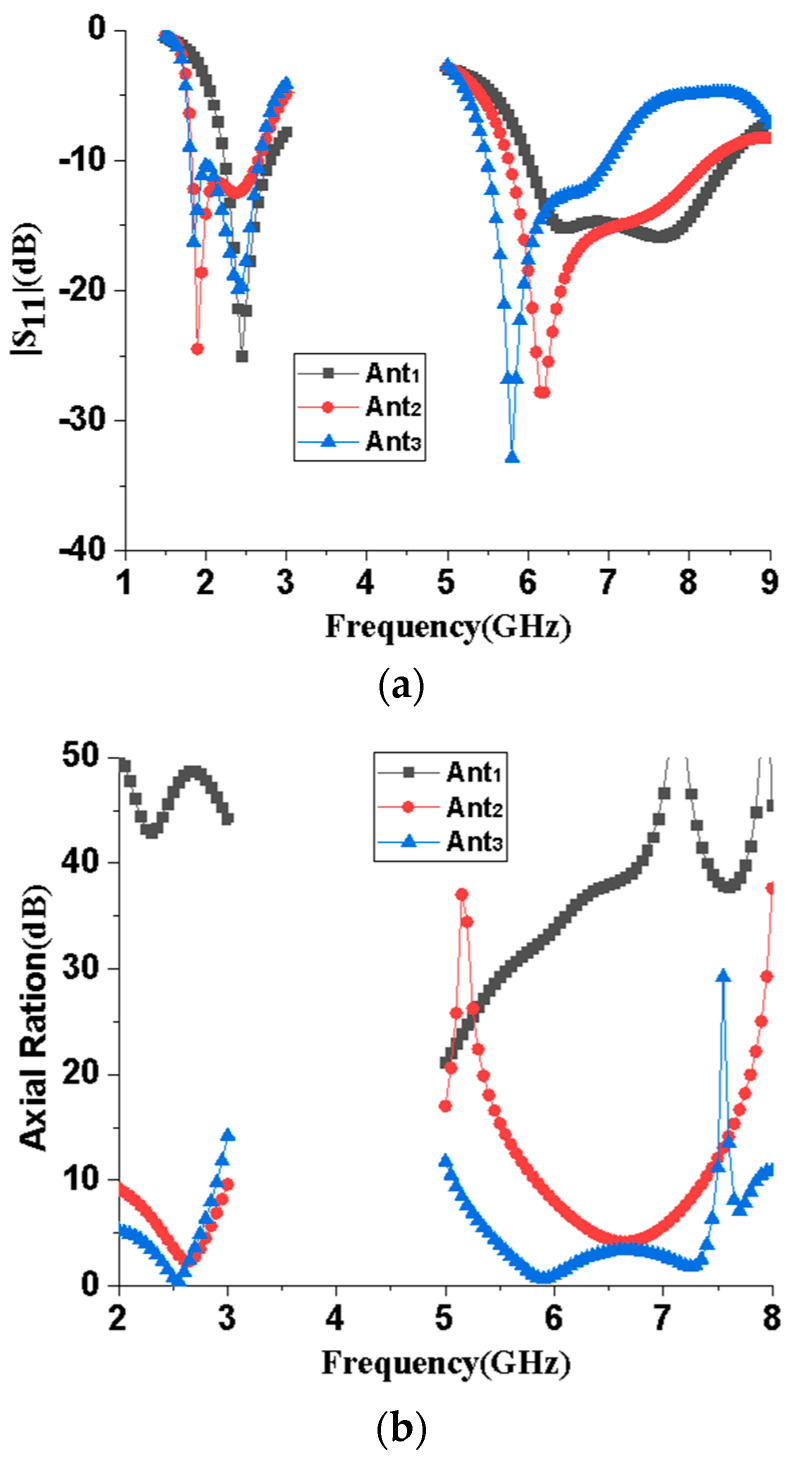
Reflection coefficients of the monopole antenna with different L3 values. (**a**) |S_11_|; (**b**) Axial Ration.

Figure 2 illustrates the antenna design process, and the three antenna prototypes are labeled as Ant1, Ant2, and Ant3, with Ant3 being the antenna proposed in this paper. As the initial design, Ant1 in Figure 2a is a CPW-fed stepped rectangular monopole antenna, whose impedance bandwidths and axial ratio bandwidths are shown in Figure 3. As can be seen from Figure 3a, Ant1 can achieve dual bands, with −10 dB impedance bandwidths of 2.24–2.78 GHz and 6.01–8.45 GHz. As shown in Figure 3b, the axial ratio of Ant1 in the 2.45 and 5.8 GHz frequency bands is much greater than 3, indicating that Ant1 is linearly polarized in both frequency bands. Ant2 adds an L-like ground branch to the right of the radiator, with impedance bandwidths of −10 dB ranging from 1.83 to 2.64 GHz and 5.76 to 8.24 GHz. Meanwhile, the axis ratio of Ant2 is close to 3 in the 2.45 and 5.8 GHz frequency bands, indicating preliminary circular polarization properties. To further improve the circular polarization characteristics of the antenna, an indirectly coupled L-shaped radiation patch was added to the left of the radiation patch of Ant2, thus forming the Ant3 proposed in this paper. Ant3 has −10 dB impedance bandwidths of 1.8–2.66 GHz and 5.48–6.98 GHz, and 3 dB axial ratio bandwidths of 2.34–2.67 GHz and 5.58–6.39 GHz, respectively. It can also be seen from Figure 3 that from Ant1 to Ant3, the operating frequency of the dual frequency bands gradually shifts towards the low-frequency direction, and the circular polarization characteristics of the dual frequency bands gradually improve.

### 2.3. CP Mechanism

In general, the ideal circular polarization is realized by two orthogonal electric field vectors with equal amplitudes and a phase difference (PD) of 90°. When the amplitudes of the two orthogonal currents are equal and the PD is 90°, an ideal current distribution is obtained. For the proposed antenna with currents in the x-axis and y-axis directions, an orthogonal current can be formed by adjusting the position and size of parasitic branches, and the current amplitude condition for circular polarization can be met, thereby achieving circular polarization.

**Figure 4:**
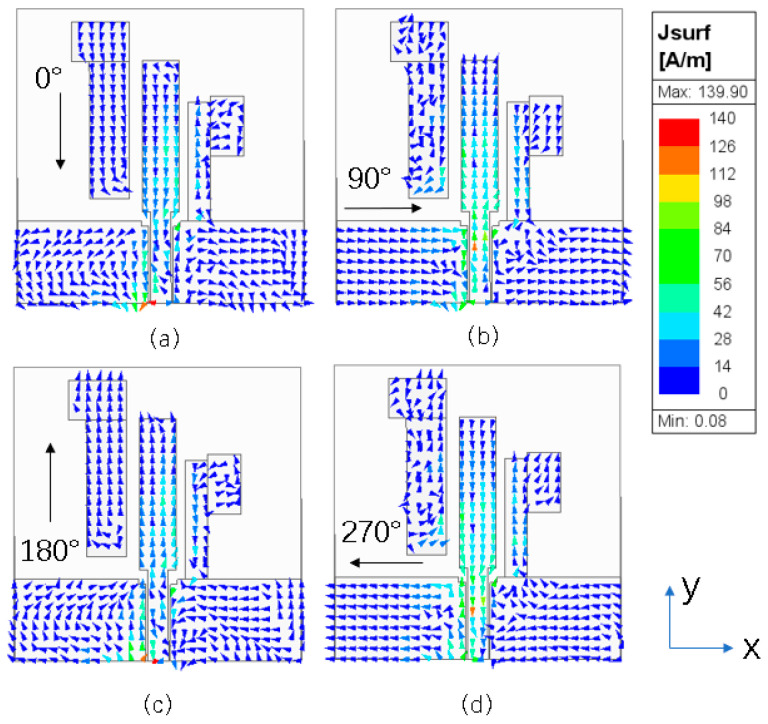
Simulated electric surface current distribution of the proposed antenna at 2.45 GHz: (**a**) 0°; (**b**) 90°; (**c**) 180°; (**d**) 270°.

**Figure 5:**
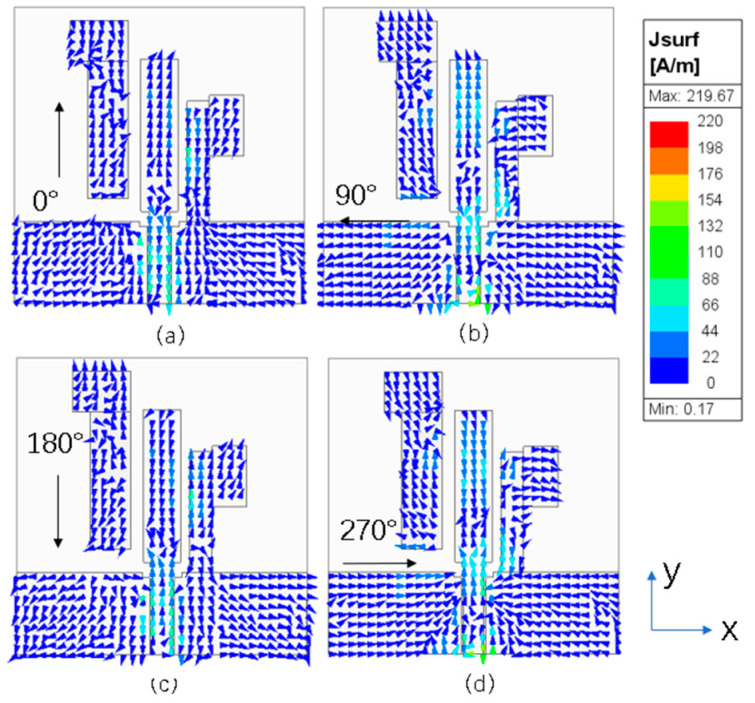
Simulated electric surface current distribution of the proposed antenna at 5.8 GHz: (**a**) 0°; (**b**) 90°; (**c**) 180°; (**d**) 270°.

To illustrate the implementation mechanism of circular polarization, Figure 4 and Figure 5 show the surface current distribution of the antenna observed from the +Z-axis at four different phases (0°, 90°, 180°, 270°) at frequencies of 2.45 and 5.8 GHz, with arrows indicating the direction of the synthesized current. Figure 4 shows the current distribution of the antenna at the 2.45 GHz frequency. At the 0° phase, the current synthesis direction is dominated by the −Y direction. At the 90° phase, the current synthesis direction is dominated by the X direction. At the 180° phase, the current synthesis direction is dominated by the Y direction. At the 270° phase, the current synthesis direction is dominated by the −X direction. When the current is in the four phases of 0° and 180°, 90° and 270°, the amplitude is equal and the direction is opposite. Since the current rotates in an anticlockwise direction, the antenna can realize the right-hand circularly polarized wave radiation at 2.45 GHz.

Similarly, Figure 5 shows the surface current distribution of the antenna at different phases at 5.8 GHz. At the 0° phase, the surface current of the antenna is synthesized in the Y direction. At the 90° phase, the surface current of the antenna is synthesized in the −X direction. At the 180° and 270° phases, the surface current has the same magnitudes but opposite phases, with respect to those at the 0° and 90° phases. Therefore, the right-hand circular polarization characteristics are realized at 5.8 GHz.

### 2.4. Parametric Analysis

**Figure 6:**
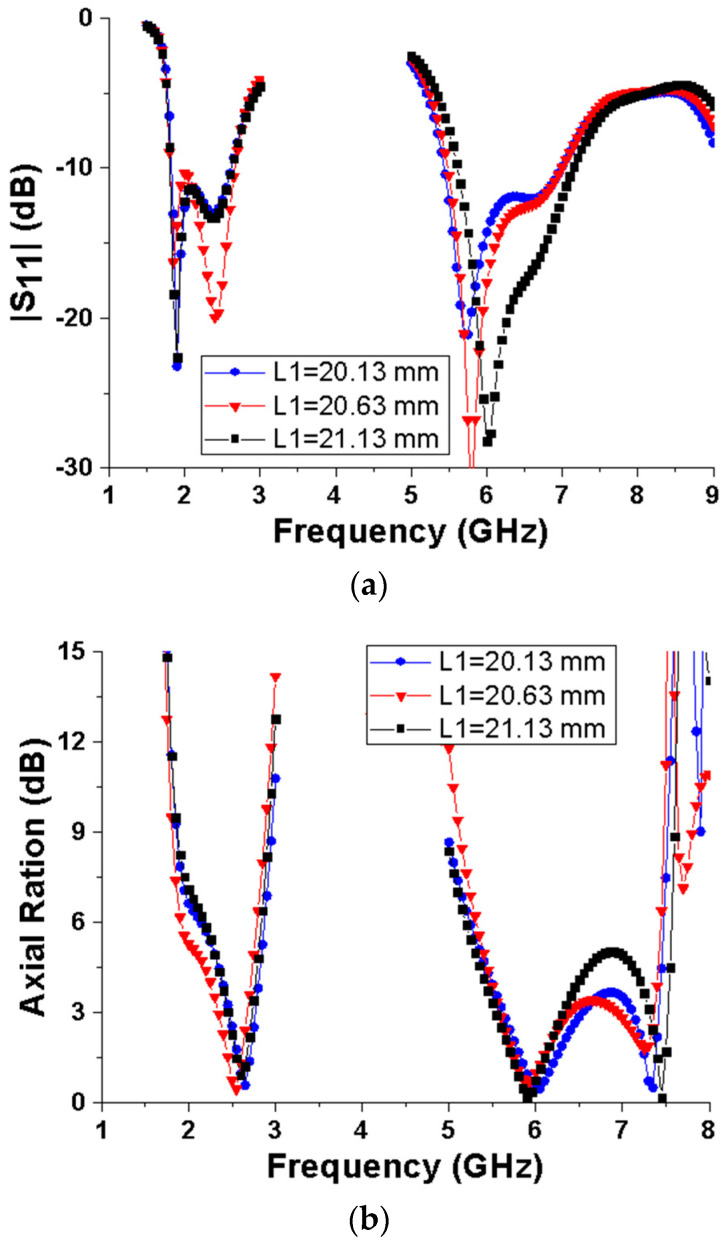
Effect of parameter L1 on antenna characteristics. (**a**) |S_11_|; (**b**) Axial Ration.

To further illustrate the influence of antenna structure parameters on antenna performance, Figure 6 shows the effect of the parasitic branching parameter L1 on S11 and AR. According to the S11 parameter in Figure 6a, as L1 increases, the −10 dB impedance bandwidth of the 2.45 GHz low-frequency band remains basically unchanged, while the 5.8 GHz high-frequency band shifts upwards and the bandwidth increases. As can be seen from the axial ratio curve in Figure 6b, when L1 increases, the 3 dB axial ratio bandwidth of the low-frequency band first increases and then decreases, while the axial ratio bandwidth of the high-frequency band decreases gradually. In addition to L1, other parameters such as L4 and L5 have a similar effect on the impedance and circularity performance of the antenna. For the sake of the brevity of this paper, no corresponding illustration is given here.

## 3. Measurement and Discussion

**Figure 7:**
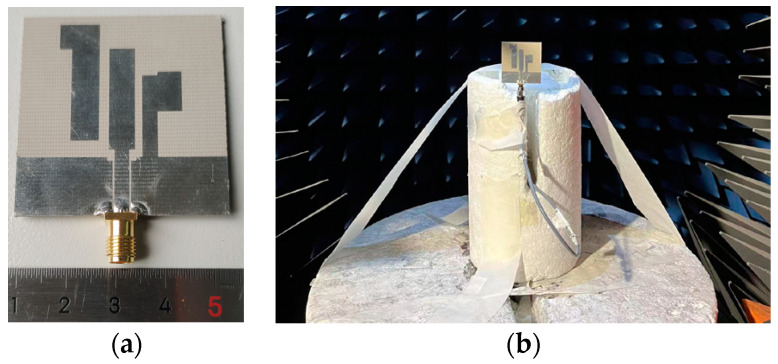
(**a**) Antenna photograph. (**b**) Pattern measurement setup.

To verify the performance of the proposed antenna, we constructed a prototype antenna and gave a measurement platform for the pattern of the antenna in the Satimo Starlab anechoic chamber, as shown in Figure 7. The relevant measurements and analyses were carried out as follows.

### 3.1. Reflection Coefficient and AR

**Figure 8:**
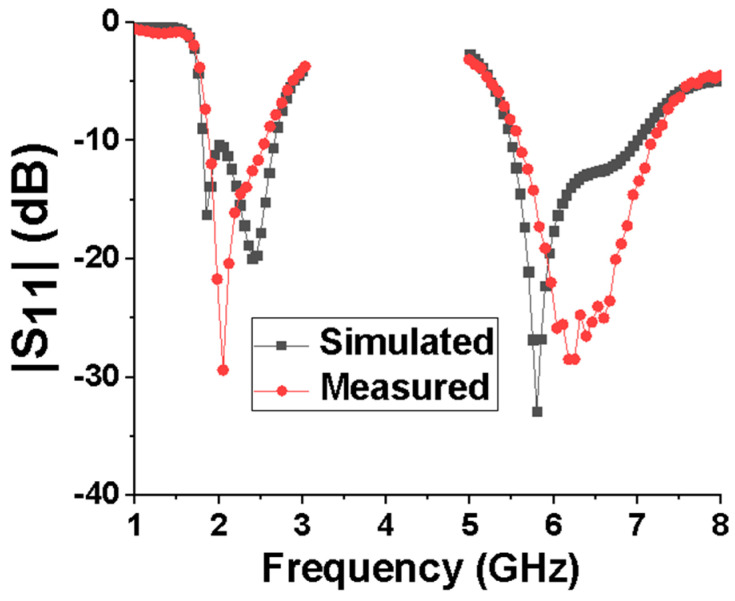
Simulated and measured reflection coefficients of the proposed antenna.

The reflection coefficient of the antenna was first measured using the vector network analyzer ZVB20 manufactured by Rhodes&Schwartz in Munich, Germany and compared with the simulation results, as shown in Figure 8. As can be seen, the measured −10 dB impedance bandwidths are 1.86–2.56 GHz and 5.58–7.2 GHz, respectively, while the simulated impedance bandwidths are 1.8–2.66 GHz and 5.48–6.98 GHz, respectively. It can be seen that the measured low-band impedance bandwidth is slightly narrower than the simulated one, while the high-band impedance bandwidth is wider.

**Figure 9:**
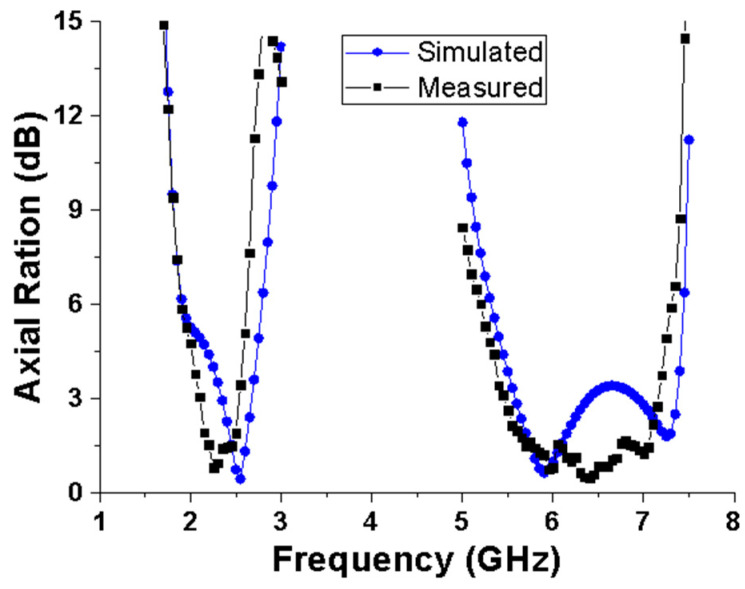
Simulated and measured AR values of the proposed antenna.

Figure 9 shows the measured 3 dB AR bandwidths, which are 2.1–2.5 GHz and 5.4–7.1 GHz, respectively, while the simulated bandwidths are 2.34–2.67 GHz and 5.58–6.39 GHz. Compared with the simulation results, the measured axial ratio bandwidth slightly deviates towards the low-frequency direction, and the overall axial bandwidth widens. Overall, the impedance and axial bandwidths of the test and simulation are basically consistent, meeting the dual-band requirements of the antenna. The deviation between the measurements and the simulation results may have been caused by manufacturing and welding errors of the antenna. It should be noted that slight deviations can be related to manufacturing errors and the effects of coaxial feeders.

### 3.2. Far-Field Characteristics

**Figure 10:**
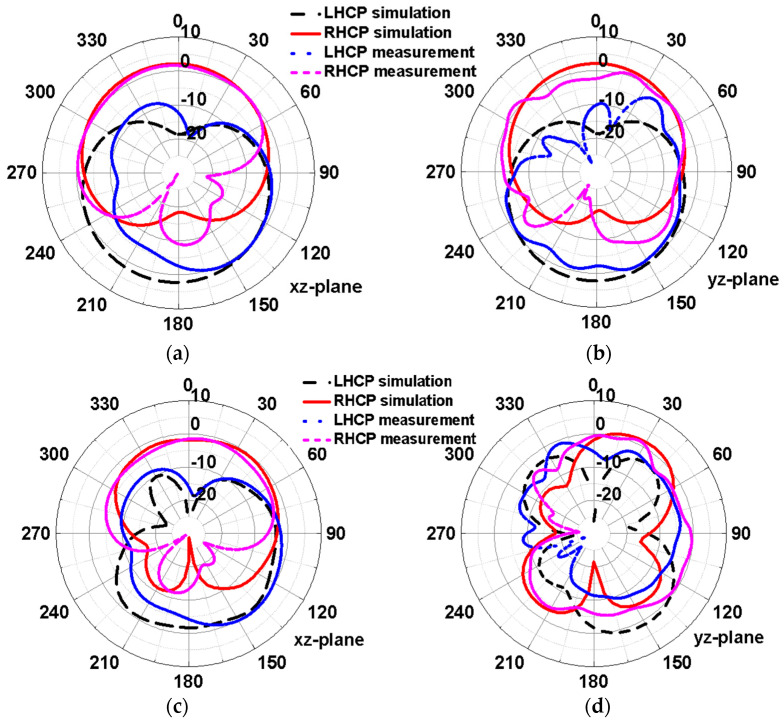
Simulated and measured radiation patterns of the integrated antenna. (**a**) in the xz-plane at 2.45 GHz; (**b**) in the yz-plane at 2.45 GHz; (**c**) in the xz-plane at 5.8 GHz; (**d**) in the yz-plane at 5.8 GHz.

Figure 10 shows the simulated and measured radiation patterns of the antenna at the 2.45 and 5.8 GHz frequency points. The radiation patterns were measured in a Satimo Starlab anechoic chamber, as illustrated in Figure 7b. As can be seen in Figure 10, the antenna exhibits bidirectional circular polarization at both 2.45 and 5.8 GHz. The dominant polarization in the dominant-view direction (+Z axis) is RHCP, while in the −Z direction, the antenna has opposite circular polarization characteristics. It can also be seen that the simulated and measured patterns of the two frequencies are basically consistent.

**Figure 11:**
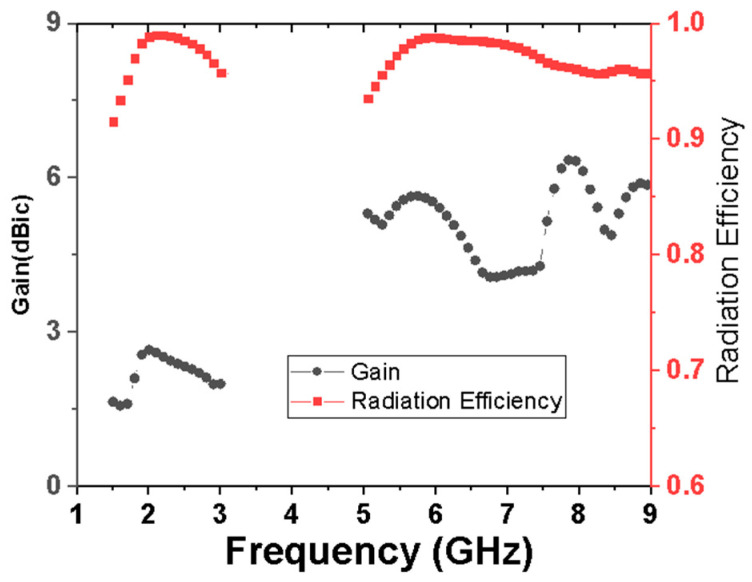
Simulated gain and radiation efficiency of the proposed antenna.

The simulated antenna gain and efficiency are shown in Figure 11. In the lower CP bands, the gain varies between 1.64 and 2.65 dBic, while in the higher CP bands, the gain varies between 4.06 and 6.36 dBic. The antenna efficiency of both bands is above 91%.

### 3.3. Wearable Performance Analysis

(1) The effect of separation on the antenna performance

**Figure 12:**
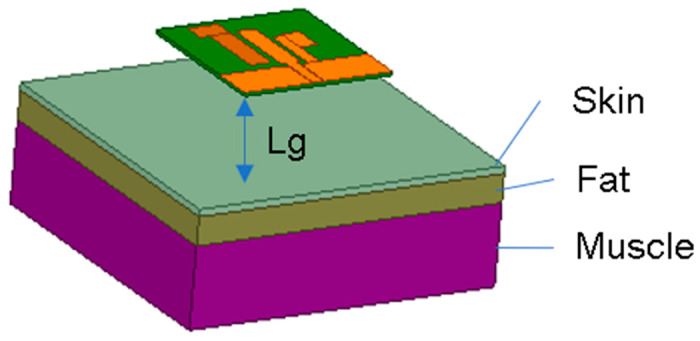
Human tissue model for the proposed antenna.

**Figure 13:**
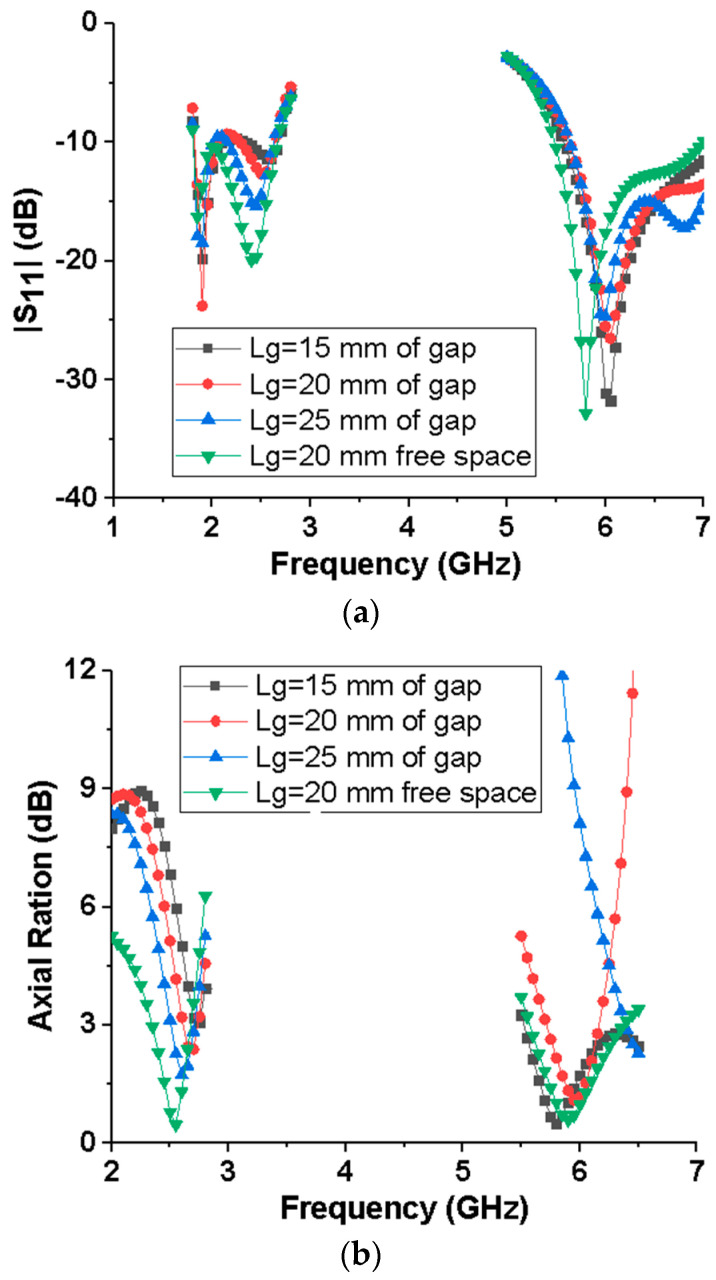
Comparison of the proposed antenna’s performance at different distances from the human body tissue model: (**a**) |S_11_|, (**b**) AR.

When the antenna is close to a human body, the human body will have some impact on the antenna’s performance. This study shows that the relative permeability of human body tissue is 1, that is, human body tissue is non-magnetic, so the study of the effect of human body tissue on the antenna only needs to consider its electrical characteristics, which are mainly composed of conduction and dielectric properties, and its dielectric properties are the most important factors affecting the antenna in the human body. The human body is composed of different tissues such as skin, fat, muscle, and bones, and the electromagnetic properties of different tissues are different, and the electromagnetic properties of the same tissues in different locations are also different. In order to analyze the effects of the human body, a three-layer human body tissue model [16] was set up in the electromagnetic simulation software, which was composed of skin, fat, and muscle, as shown in Figure 12. The size of the human body tissue model was 90 mm × 90 mm × 33 mm, consisting of 2 mm thick skin, 8 mm thick fat, and 23 mm thick muscle tissue, and its electrical characteristics parameters were sourced from references [16,17]. The effect of human body tissue on antenna performance at different separation distances between the antenna and human body tissue was the main factor we considered. Figure 13 shows the effect of the gap on the S_11_ and AR of the antenna when the antenna was separated from the human body tissue by 15 mm, 20 mm, and 25 mm, respectively, and compares the performance of the antenna with that in free space. As can be seen in Figure 13a, the S_11_ of the antenna in both the 2.45 GHz and 5.8 GHz bands deteriorates, and the widths of both bands become narrower, but their −10 dB band still covers the 2.45/5.8 GHz ISM dual bands (2.400–2.4835 GHz and 5.725–5.875 GHz). For the axial ratio performance, as shown in Figure 13b, when the separation between the antenna and human body tissue decreases, the 3 dB AR band in the 2.45 GHz frequency band moves towards high frequencies, while the 3 dB AR band in the 5.8 GHz frequency band moves towards low frequencies. It can also be seen from Figure 13 that the closer the antenna and the human body tissue are, the greater the effect of the human body tissue platform on the S_11_ and AR performance of the antenna. When the antenna works in free space, it can be regarded that the working state of the antenna is sufficiently away from the human body. To achieve better performance when the separation between the antenna and the human body is small, the human body tissue platform should be considered when designing antennas.

(2) Evaluation of wearable safety

With the wide application of wireless electronic devices, human health problems caused by radiation from wearable antennas have attracted more and more attention from researchers. At present, the effect of antenna radiation on human body tissues is studied by analyzing the ability of human body tissues to absorb electromagnetic waves, and the degree of harm to the human body is measured by the electromagnetic power absorbed by human body tissues per unit mass, namely the specific absorption rate (SAR). The formula for calculating the SAR is given in Equation (1):

(1) $$SAR=\frac{\sigma E^{2}}{\rho }$$

where σ is the conductivity of the human tissue in S/m, *E* is the effective electric field in V/m, and ρ is the density of human tissue in kg/m^3^.

**Figure 14:**
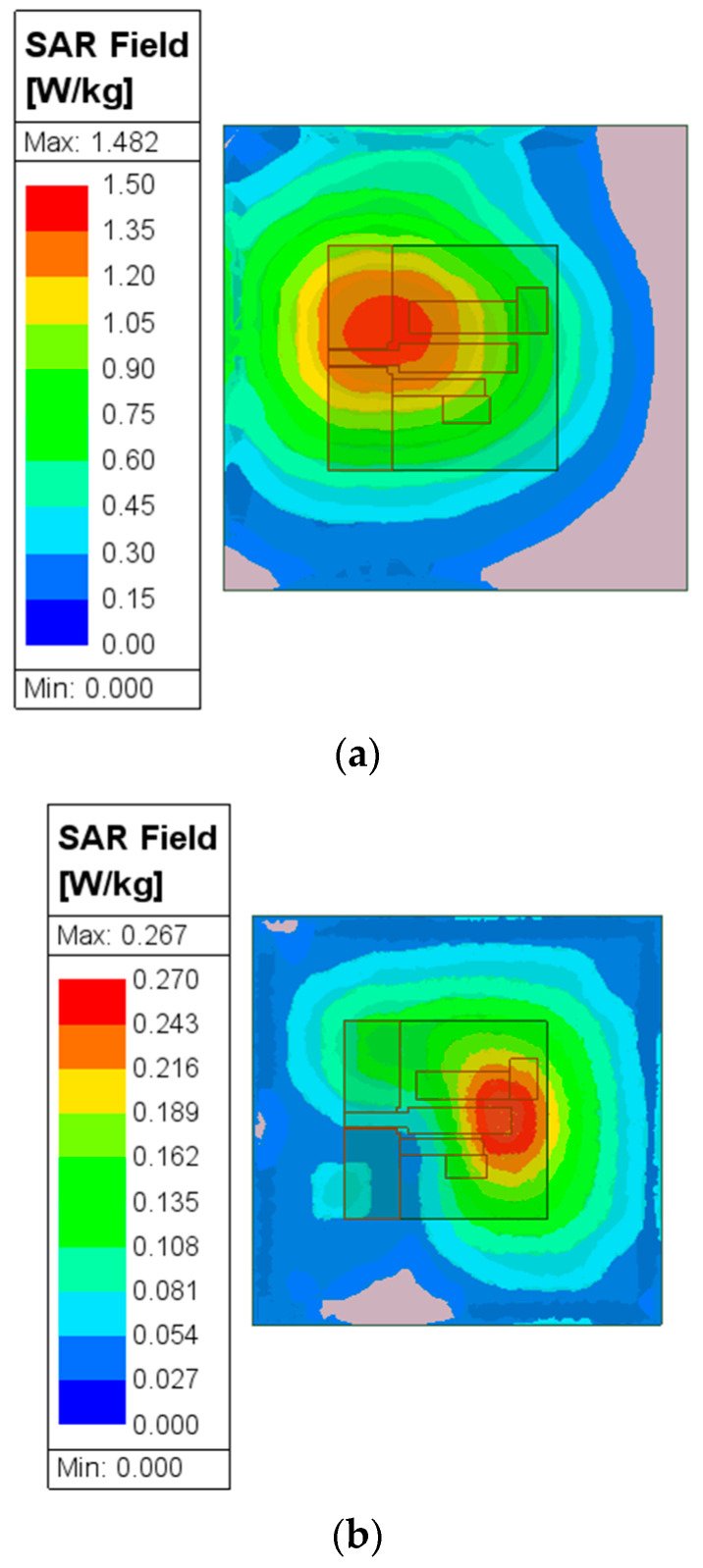
SAR level of the proposed antenna at (**a**) 2.45 GHz; (**b**) 5.8 GHz.

To evaluate the safety performance of proposed wearable antennas, the specific absorption rate (SAR) value of the antenna near the human body is usually evaluated. Here, the human tissue model is also derived from the literature [16,17]. According to the IEEE C95.1 standard [18], in order to ensure sufficient safety for the human body when exposed to electromagnetic radiation in an electromagnetic environment, the SAR value of the antenna should be lower than the upper limit of the standard. When the input power is 0.1 W, the maximum SAR value that the human body can withstand under the 1 g standard should be less than 1.6 W/kg. The distance between the human body tissue model and the antenna is about 20 mm, and the input power is 0.1 W. Figure 14 shows the SAR distribution of human body tissues at frequencies of 2.45 GHz and 5.8 GHz, with SAR values of 1.48 and 0.27 W/kg, respectively, which are less than 1.6 W/kg and meet the US standards. Therefore, the proposed antenna meets the requirements for human wearability.

(3) Practical performance of the antenna at different human body positions

**Figure 15:**
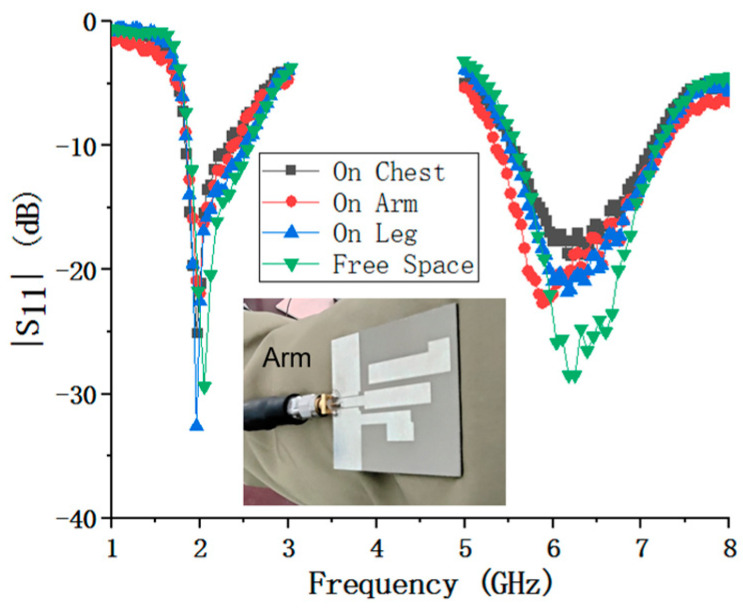
Measured S_11_ of the proposed antenna at different human body positions.

In order to verify the practical performance of the wearable antenna at different positions of the human body, the antenna was measured on the chest, arms, and legs using the aforementioned vector network analyzer, and the reflected coefficients measured are shown in Figure 15. Although the resonance depth and center frequency show some variations at different human body parts, the impedance bandwidth of S_11_ < −10 dB can still cover the 2.4 and 5.8 GHz bands, which means that the antenna maintains a good impedance match at different positions on the human body.

The results indicate that the proposed antenna has good wearable performance. In specific application scenarios, antenna performance can be further improved by considering various influencing factors in antenna designs.

### 3.4. Performance Comparison

Table 2 shows a comparison of the proposed antenna with the existing wearable CP antenna. As can be seen from Table 2, except for the dual-band circularly polarized wearable antenna in Ref. [15], the other antennas are single-band circularly polarized wearable antennas. Compared with single-band circularly polarized antennas, dual-band circularly polarized antennas have the characteristics of multi-functionality and a large capacity. The antenna in Ref. [15] is composed of a dual-band monopole radiator and a polarized rotating artificial magnetic conductor (PRACM), and dual-circular polarization is achieved by both. The antenna structure is relatively complex. In addition, the antenna has a narrow dual-band impedance bandwidth (IMBW) and axial ratio bandwidth (ARBW). However, the structure of the dual-band circularly polarized antenna proposed in this work is relatively simple, and the impedance bandwidth and axial ratio bandwidth are better than those reported in the literature [15], and the plane size is also smaller. Compared with the existing wearable circularly polarized antennas, the proposed antenna has better performance.

## 4. Conclusions

In this paper, a new miniaturized dual-band circularly polarized antenna is proposed. Due to the addition of indirectly coupled L-shaped parasitic patches and L-like ground parasitic patches on both sides of the monopole radiation patch, the antenna can achieve circular polarization in the 2.45 and 5.8 GHz ISM dual frequency bands. The impedance bandwidth, axial ratio bandwidth, radiation pattern, and specific absorption rate of the antenna were analyzed. The proposed antenna has characteristics such as a miniaturized size, dual bands, circular polarization, and broadband properties, making it suitable for application in wearable systems in wireless body area networks.

## Figures and Tables

**Table 1:** The dimensions of the proposed antenna.

Symbol	Value (mm)	Symbol	Value (mm)	Symbol	Value (mm)
W	43.26	W3	8.73	hs	1.016
L	44.59	L3	6.06	h1	0.89
W0	3.15	W4	3.31	h2	0.79
L0	13.86	L4	18.14	g1	1.21
W1	5.57	W5	9.08	g2	1.10
L1	22.86	L5	5.17	S	0.3
W2	5.99	Lg	19.89	Lg1	14.26
L2	20.63				

**Table 2:** Comparison of the proposed antenna with existing wearable CP antennas.

Ref.	f0 (GHz)	IMBW	ARBW	Min S11 (dB)	Polarization	Dimensions (mm^3^)	Peak Gain (dBic)	EFF	SAR (W/kg)
[10]	5.8	6.89%	1.38%	−37	CP	27 × 23 × 1.5	6.0	-	1.02
[12]	5.8	109%	98.5%	−20	CP	25 × 30 × 1	4.4	82%	1.02
[14]	1.575/2.45	7.6%/5.5%	10.3%	−25/−14	CP/LP	85.5 × 85.5 × 5.6	1.98/1.94	-	0.086/0.087
[15]	3.5/5.8	11.7%/9.1%	2.0%/ 8.2%	−38/−25	CP/CP	62 × 62 × 5.5	6.6/7.2	57.5%/60.25	0.128/0.043
This work	2.45/5.8	38.6%/24.1%	13.3%/ 13.5%	−20/−33	CP/CP	48.7 × 42.1 × 1.0	2.65/6.36	91%/ 92%	1.48/0.27

## Data Availability

The original contributions presented in the study are included in the article. Further inquiries can be directed to the corresponding author.
